# X-IGA Used for Orthotropic Material Crack Growth

**DOI:** 10.3390/ma17153830

**Published:** 2024-08-02

**Authors:** Mohammed Berrada Gouzi, Ahmed El Khalfi, Sorin Vlase, Maria Luminita Scutaru

**Affiliations:** 1 Faculty of Science and Technology, Sidi Mohamed Ben Abdellah University, Fez 30000, Morocco; ahmed.elkhalfi@usmba.ac.ma; 2 Department of Mechanical Engineering, Faculty of Mechanical Engineering, Transylvania University of Brasov, B-dul Eroilor 29, 500036 Brasov, Romania; 3 Romanian Academy of Technical Sciences, B-dul Dacia 26, 030167 Bucharest, Romania; lscutaru@unitbv.ro

**Keywords:** unidirectional composite material, crack growth, extended isogeometric analysis, stress intensity factor, energy integral method, Stroh’s formula, extended finite element method

## Abstract

In this paper, we propose a new approach for numerically simulating the growth of cracks in unidirectional composite materials, termed extended isogeometric analysis, evaluating the maximum stress intensity factor and T-stress. To validate our approach, we used a small anisotropic plate with two edge cracks, beginning with formulating the governing equations based on the energy integral method, Stroh’s Formula, and the Elastic Law describing the behaviour of anisotropic materials, while considering boundary conditions and initial states. A MATLAB code was developed to solve these equations numerically and to post-process the tensile stress and the stress intensity factor (SIF) in the first mode. The results for the SIF closely match those obtained using the extended finite element method (X-FEM), with a discrepancy of only 0.0021 Pa·m^0.5^. This finding underscores the credibility of our approach. The extended finite element method has demonstrated robustness in predicting crack propagation in composite materials in recent years, leading to its adoption by several widely used software packages in various industries.

## 1. Introduction

The integrity of a mechanical product requires numerical validation when the calculation of the tensile stress and the stress intensity factor around critical zones, specifically, cracked ones, is essential for predicting damage [1]. That is why, in the field of composite materials, researchers have developed several approaches, such as the X-FEM, which provides efficient results for anisotropic materials. However, the calculation time remains long and CPU-intensive. Our paper proposes the use of the X-IGA technique to reduce the calculation time and improve the accuracy of the numerical results.

X-IGA is particularly helpful for breaking down mechanical systems into components connected by nodes and establishing boundary conditions [2,3], which collectively form a mesh that approximates the geometry under study [4]. Extended finite element approaches, on the other hand, are useful when singularities are present and the conventional method is not suitable. To overcome such challenges, these techniques amplify concentrated stresses using the Heaviside function and enhance crack edges.

Because of the unique characteristics of non-uniform rational B-splines (NURBS) [5,6] the importance of isogeometric analysis (IGA) has grown significantly in recent years. This methodology provides a curved approximation, which is very useful for properly representing the curved geometries present in the system being studied. In addition, the flexibility of NURBS makes it easier to approximate partial derivatives for functions that express the geometric properties of the system, which improves IGA’s analytical capabilities and precisely captures complex geometric aspects.

Similar to the finite element method (FEM) [7], the isogeometric analysis (IGA) approach first encountered difficulties while managing singularities. Nevertheless, as the literature [8] shows, more recent developments have resulted in the creation of extensions like X-IGA and the X-FEM, which have improved capabilities for handling crack-related problems. Notably, by using fewer computational grids than the X-FEM, X-IGA offers notable gains in terms of computing efficiency. Specifically, X-IGA, which is comparable to the X-FEM, applies the concepts of IGA to mechanical fracture propagation and allows for the precise calculation of stress, strain, and the maximum stress intensity factor (SIF) using comparable computer models and equations.

As per papers [8,9], S. Montassir demonstrates that X-IGA and the X-FEM have been highly effective in providing a numerical estimation of the stress intensity factor when compared to experimental data. Therefore, this paper can further delve into this analysis, particularly in the context of anisotropic materials, and validate the consistency of the results by verifying convergence between the two methods.

This paper is composed of five sections, with the aim of presenting the main idea of the article in an orderly fashion. Section 1 is devoted to introducing the main idea and projecting towards the content of the article.

The purpose of the Section 2 is to clarify our approach. It gives a brief introduction to isogeometric analysis (IGA), clarifies the meaning of non-uniform rational B-splines (NURBS) basis functions, and shows how two-dimensional numerical simulations can use NURBS basis functions in place of Lagrange basis functions. Our particular study situation is mathematically described in Section 3, taking into account the part’s mechanical properties, which deviate greatly from those of isotropic materials [10]. The main system of equations for static elastic deformation is then formulated, describing displacement near the crack and the stress intensity factor, respectively, with the help of Stroh’s formula [11] and the Energetic Method [12,13,14,15,16,17,18,19,20,21,22,23,24,25,26].

The numerical simulation of the studied model is presented in the Section 4. It starts with input data on the geometry and material properties of the portion that is being studied, which is fixed on one side and exposed to tensile stress on the other, as shown in Section 3 The authors pre-processed the same input data for our suggested X-IGA and X-FEM techniques, and then we compared the findings and held a discussion.

A summary of our findings is provided in the conclusion of this article, which emphasizes the efficiency of X-IGA for both isotropic and unidirectional composite anisotropic materials. Compared to the X-FEM, X-IGA simplifies the process by going straight from Computer-Aided Design (CAD) to the solving steps [6]. It also emphasizes how NURBS basis functions can represent geometry with lowest possible stiffness matrix dimensions [27]. The crucial issue, “Will IGA eventually supplant the FEM in all structural simulation applications?” is addressed in the final paragraph.

## 2. Methodology

### 2.1. Overview of IGA and X-IGA 

Currently, isogeometric analysis is one of the most-employed methods for numerical calculations and the simulation of structural behaviour. This is primarily due to the effectiveness of NURBS as a prominent approach for handling curved geometries. By leveraging the basic functions of this methodology instead of Lagrange basis functions, we can achieve more precise results while reducing the grid count and optimizing solving time.

Similar to the X-FEM, X-IGA serves as an extension of IGA tailored for studying mechanical crack propagation. By applying consistent logic and formulas, we can derive approximate values for stress, strain, and the maximum stress intensity factor (SIF).

### 2.2. IGA Concept

The knot vector is {*ξ*} = {*ξ*_1_, …, *ξ*_*n*+*p*+1_}. The polynomial function of order P, *N*_*i*_, serves as the B-spline basis component [28,29] and is written in formal notation:

(1) $$N_{i,0}(\xi )=\left\{\begin{array}{c} 1\;\mathrm{if}\;\;\xi_{i}\leq \xi \leq \xi_{i+1}\;\\ 0\;\mathrm{in}\;\mathrm{the}\;\mathrm{other}\;\mathrm{cases} \end{array}\right.$$

and for P > 0,

(2) $$N_{i,p}\left(\xi \right)=\frac{\xi -\xi_{i}}{\xi_{i+p}-\xi_{i}}N_{i,p-1}\left(\xi \right)+\frac{\xi -\xi_{i+1}}{\xi_{i+p+1}-\xi_{i+1}}N_{i-1,p-1}\left(\xi \right)$$

Using the last formula to construct surfaces based on the control point *B*_*i*,j_, there is another function for the second direction, named *M*_*j*,*q*_, where the order here is q and the knot vector is {*η*} = {*η*_1_, …, *η*_n_}. The curve is defined by the following:

(3) $$M_{j,q}\left(\xi \right)=\frac{\eta -\eta_{i}}{\eta_{i+p}-\eta_{i}}M_{i,p-1}\left(\xi \right)+\frac{\eta -\eta_{i+1}}{\eta_{i+p+1}-\eta_{i+1}}\eta_{i-1,p-1}\left(\xi \right)$$

### 2.3. NURBS for Two Dimensions

The difference between NURBS and B-spline functions is the introduction of a set of the n × n positive elements *w*_*i*_, called weight. According to citations in articles [20,28], the rational basis function is as follows:

(4) $$R_{i,j}(\xi ,\eta )=\frac{N_{i,p}\left(\xi \right)M_{j,q}\left(\eta \right)w_{i,j}}{\sum_{a=1}^{n}\sum_{b=1}^{n}N_{a,b}\left(\xi \right)M_{a,b}({\eta )\;w}_{a,b}}$$

The curve’s formula is as follows:

(5) $$C\left(\xi ,\eta \right)=\sum_{i=1}^{n}\sum_{j=1}^{n}R_{i,j}(\xi ,\eta )\;\;B_{i,j}$$

## 3. Mathematical Formulation

### 3.1. Elastic Behaviour Law for Anisotropic Material

In case of 2D studies, the stress–strain relation for anisotropic material can be expressed by the following:

(6) $$\left[\begin{array}{c} \sigma_{x}\\ \sigma_{y}\\ T_{xy} \end{array}\right]=\left[\begin{array}{ccc} \frac{E_{x}}{1-\nu_{xy}\nu_{yx}\;} & \frac{\nu_{xy}E_{y}}{1-\nu_{xy}\nu_{yx}\;} & 0\\ \frac{\nu_{xy}E_{y}}{1-\nu_{xy}\nu_{yx}\;} & \frac{E_{y}}{1-\nu_{xy}\nu_{yx}\;} & 0\\ 0 & 0 & G_{xy} \end{array}\right]\left[\begin{array}{c} \varepsilon_{x}\\ \varepsilon_{y}\\ \Upsilon_{xy} \end{array}\right]$$

The Coulomb modulus $G_{xy}$ is defined by the following:

(7) $$G_{xy}=\frac{E_{x}\;E_{y}}{E_{x}+E_{y}+2E_{y}\nu_{xy}\;}$$

**Figure 1:**
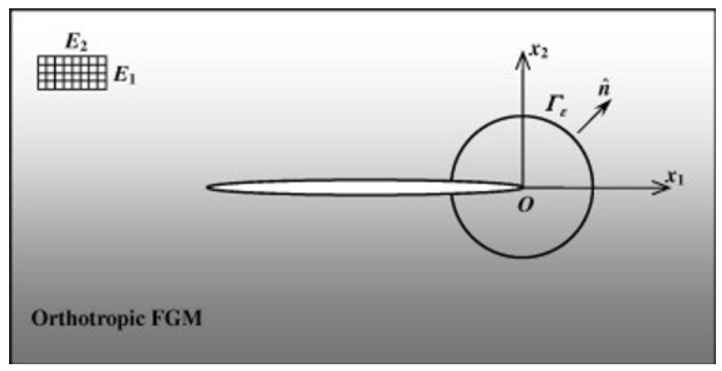
A general anisotropic plate in tension for a plane of coordinate (X,Y) with the polar component of the crack (r, θ).

The symmetry of the elasticity matrix has been satisfied because $\nu_{xy\;}E_{y}=\nu_{yx\;}E_{x}$, where $\nu_{xy\;}$ $\nu_{yx\;}$ are Poisson coefficients of the material in the case of the 2D anisotropic one, so automatically $\nu_{xy}\;\neq \;\nu_{yx}$ (Figure 1).

For the equilibrium stat, the equation is as follows:

(8) $$\left\{\begin{array}{c} Div\left(\sigma_{ij}\right)=0\\ \varepsilon_{ij}=\frac{1}{2}\;(\frac{\partial U_{i}}{\partial x_{j}}+\frac{\partial U_{j}}{\partial x_{i}}) \end{array}\right.$$

### 3.2. Expression of the Displacement near the Crack

Stroh’s formula ensures that the displacement near a crack in an anisotropic 2D plate is the real part of the displacement’s asymptote, and it can be calculated using the following relation:

(9) $$U_{i}(r,\theta )=\sqrt{\frac{2}{\pi }}Re(K_{\alpha }A_{im}{B_{m\alpha }}^{-1}(\sqrt{r(\cos\left(\theta \right)+\mu_{m}sin(\theta )})$$

where i, m = 1, 2 et α = I, II the deformation in modes I and II. Using the Einstein indices, the formula of stress can be expressed by the following:

(10) σij=−1j12π Re(KαBimBmα−1δjl+μmδj2(r(cos⁡θ+μmsin⁡(θ)))

Using *δ*_*jk*_, as the Kronecker delta and *μm* representing distinct complex numbers with the imaginary part from Equation (11),

*a*_11_*μ_m_*^4^ − 2 *a*_16_
*μ_m_*^3^ + (2 *a*_12_ + *a*_66_)*μ_m_*^2^ − 2 *a*_26_*μ_m_* + *a*_22_ = 0 (11)

### 3.3. The SIF Using the Interaction Energy Integral Method

As a reliable and efficient method, the authors purposefully chose to apply the energetic integral method in this investigation. The integral method of interaction energy provided an older approach based on an energetic viewpoint that was very helpful in complex situations arising in anisotropic materials. Through the application of this approach, they aimed to tackle various issues presented by the behaviour of these materials, such as directional dependence and heterogeneity. Furthermore, the interaction energy integral approach offered an extensive framework for examining crack propagation and related stress fields in anisotropic materials [11]. This technique made it easier to calculate important quantities, such as the stress intensity factor (SIF), which was essential for estimating the likelihood of fracture propagation and structural failure. In addition, this method provided flexibility and adaptability based on energy balance, making it an excellent choice for managing the transformation of elastic energy into displacement energy. Researchers could thereby control crack growth and capture the complex interactions between forces and displacement within the material structure. This capability was particularly crucial when working with anisotropic materials, where conventional analytical techniques may not fully capture material behaviour.

Overall, this methodological approach aimed to improve the breadth and accuracy of studying orthotropic materials, advancing our understanding of their mechanical behaviour. The expression of this energy is given by the following:

(12) $$J=\iint (W\delta_{1j}-\sigma_{ij}U_{i,1})n_{j}ds,$$

where *i*, *j* = 1, 2 denotes our two-dimensional problem, s is an arbitrary closed curve surrounding the crack directed by the normal vector n, and w represents the stress–strain elastic energy defined as

(13) $$w=\frac{1}{2}\sigma_{ij}:\varepsilon_{ij}.$$

By superposing the true and the auxiliary states, the energy can be decomposed as

(14) $$J^{\left(1+2\right)}=J^{\left(1\right)}+J^{\left(2\right)},$$

where

- ^(1)^ denotes the integral in the true state over an arbitrary area around the crack.
- ^(2)^ denotes the integral in the auxiliary state, chosen to coincide with the crack tip asymptotic field. It satisfies both the equilibrium and the traction-free boundary condition on the crack surface.

For the elastic anisotropic solid under mixed mode loading, the *J* integral is also expressed as

(15) $$J=K^{T}LK$$

where

K = [*K*_*I*_   *K*_*II*_] and L = Re(iA*B*^−1^) ∆. (16)

Referring to papers [6,7,27], the stress intensity factor (SIF) of anisotropic materials is given by the following:

(17) $$\left[K_{II}\;\;K_{I}]=\sqrt{\frac{\pi }{8r}}{R_{e}}^{-1}\left(iAB^{-1}\right)[U_{1}U_{2}\right]$$

The crack tip enrichment function in matrix form [11] is as follows:

(18) $$F_{\alpha }\left(r,\theta \right)=\sqrt{r}\left\{R_{e}\left[\frac{B^{-1}A_{1}\beta }{B^{-1}A_{2}\beta }\right]\right\}$$

where

*A*_1_ = [*A*_11  12_] and *A*_2_ = [*A*_21_   *A*_22_]

In an anisotropic medium, the matrices *A* and *B* depend on the properties of the anisotropic materials and are independent of the coordinate system.

The standard DOFs are represented by *u*_*i*_, $a_{j}$ represents the crack faces, and, finally, the enrichment DOFs for tip singularities are represented by $b_{k}^{\beta }$.

### 3.4. X-IGA Formulation for Linear Elastic Fracture

**Figure 2:**
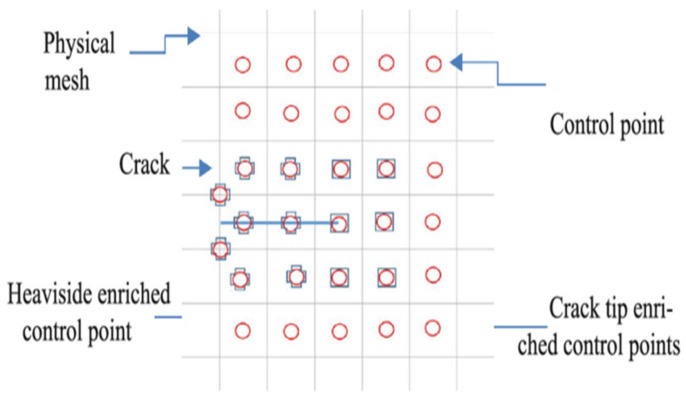
Enrichment nodes of X-IGA.

Due to the intrinsic effectiveness of isogeometric analysis (IGA) in handling singularities, X-IGA is a powerful enhancement. The key strategy involves adding enrichment functions designed specifically for crack analysis to the IGA model, alongside the use of the Heaviside function, as described in the cited paper [30]. Thanks to these capabilities, X-IGA can accurately compute, capture, and study crack behaviour as well as calculate the stress intensity factors (SIFs) and forces around cracks, as illustrated in Figure 2.

Far from the crack position, where there is no enrichment, the displacement of the control points can be expressed as

(19) $${U_{i}}^{IGA}=\sum_{1}^{N1}{\Phi_{i}U}_{i}$$

$\Phi_{i}$ denotes the shape function, referring to the univariate NURBS basis function. The expression of the displacement around the crack utilizes the Heaviside function, as written in the following formula:

(20) $${U_{i}}^{HEAVISIDE}=\sum_{1}^{N2}N_{i}H(x)a_{i}$$

For the knots enriched near the crack front, the authors used the following formula:

(21) $${U_{i}}^{Nearcrack}=\sum_{i=1}^{NTIP}\sum_{\alpha }N_{i}F_{\alpha }(x){b^{\alpha }}_{i}$$

where

- H(x) is the Heaviside function defined as H(x) = $\left\{\begin{array}{c} +1\;above\;crack\\ -1\;below\;crack \end{array}\right.$.
- N1, N2, and NTIP represent the number of standard IGA elements, the number of elements enriched with the Heaviside function, and the number of knots enriched near the crack front, respectively. F(x) denotes the enrichment function near the crack front. The total displacement is now expressed as follows:
  (22) $$U^{h}\left(\xi \right)=\sum_{i=1}^{Nenr}B_{i}(\xi )u_{i}+\sum_{j=1}^{Nd}\left\{H\left(\xi \right)-H(\xi_{j})\right\}a_{j}+\sum_{k=1}^{Nt}B_{k}\left(\xi \right)\sum_{\beta =1}^{4}\left[F_{\beta }\left(\xi \right)-F_{\beta }\left(\xi_{k}\right)\right]b_{k}^{\beta }$$

### 3.5. IGA Stiffness Matrix Construction

Considering a physical domain named Ω, bounded by ℾ, a distinction is made between two kinds of force distributions: volume density and surface density, located on ℾ. The equilibrium static equation is written as

∮ (*u*): *D*: (*δu*)Ω = ∬_ℾ_
*t δu*
*d* ℾ + ∭_Ω_
*b*
*δu*
*d*Ω (23)

where D refers to the elasticity matrix and b and t denote the body force distribution and boundary stress, respectively. By interpolating the displacement with NURBS basis functions and using the Bubnov–Galerkin method [31], where the same shape functions Φ*i* are used for both u and δu, the formula is as follows:

(24) $$\left\{\begin{array}{c} u\left(x\right)=\sum_{i=1}^{N1}\Phi_{i}\left(\xi \right)u_{i}\\ \delta u\left(x\right)=\sum_{i=1}^{N1}\Phi_{i}\left(\xi \right){\delta u}_{i} \end{array}\right.$$

where δ*u*_*i*_ denotes the nodal displacement variation. 

The derivative of the shape function is defined in the physical space as a function of the parametric space representation:

(25) $$\left[\Phi_{i,x}\;\;\;\;\;\;\;\;\;\Phi_{i,y}\right]=\left[\Phi_{i,\xi }\;\;\;\;\;\;\;\;\;\Phi_{i,\eta }\right]\left[\begin{array}{cc} \xi_{,x} & \xi_{,y}\\ \eta_{,x} & \eta_{,y} \end{array}\right]$$

In the case of two-dimensional simulation, B is given by the following:

(26) $$\left[B\right]=\left[\begin{array}{c} \Phi_{i,x}\;\;\;\;\;\;\;\;\;0\\ \;\;\;\;0\;\;\;\;\;\;\;\;\;\;\;\;\Phi_{i,y}\\ {\;\;\;\;\Phi }_{i,y}\;\;\;\;\;\;\Phi_{i,x} \end{array}\right]$$

With the substitution of Equation (22), we can derive the following discrete set of equations:

(27) $$\left\{\begin{array}{c} {}[K]\;\{u\}=\{f\}\;\;\;\;\;\;\\ Ki,j=∭Ω\;BiT\;D\;Bj\;dΩ\;\\ \;fi=\iint ℾ\;\Phi i\;t\;d\;ℾ+∭Ω\;\Phi i\;b\;d\;Ω \end{array}\right.$$

where i = 1, 2, 3 and j = 1, 2.

**Figure 3:**
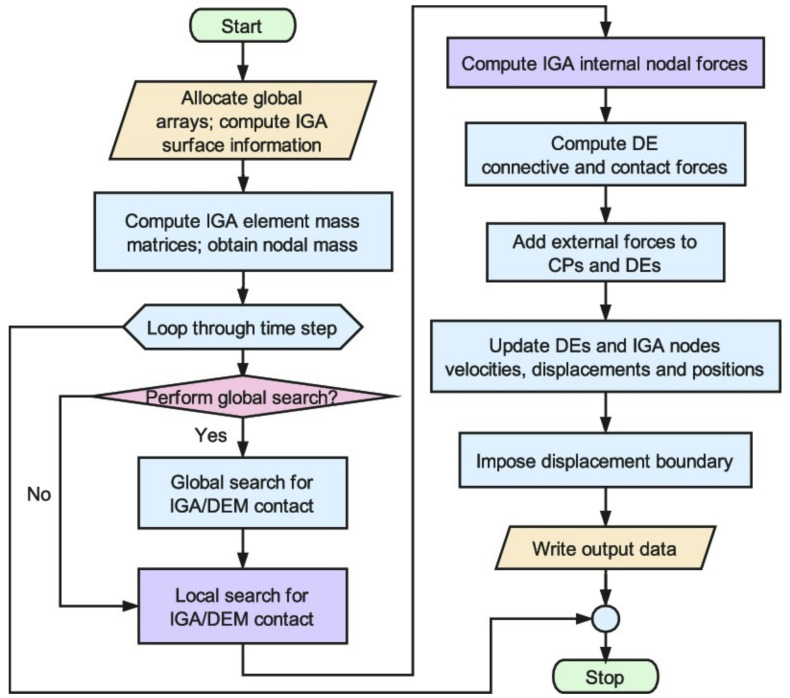
An IGA workflow simulation.

An optimized workflow can be used to create an efficient algorithm with a MATLAB code to simulate models using IGA (Figure 3). 

## 4. Simulation and Discussion

### 4.1. Geometry and Material Inputs Data

In this study, a 2D plate made of composite unidirectional orthotropic material was used to evaluate the efficiency of X-IGA in calculating the maximum stress intensity factor (SIF) and tensile stress for the first mode. Table 1 and Table 2 below contains details about the geometry.

The material used was Epoxy Carbon UD 230 GPa prepreg, described in Table 2.

### 4.2. X-IGA and X-FEM Pre-Processing

**Figure 4:**
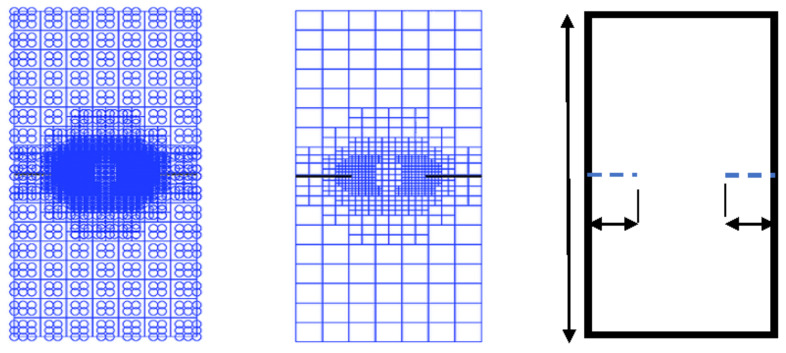
NURBS representation of the 2D plate mesh (h-refinement) p = q = 3.

For the identical case study with the same plate geometry, crack length, and material, we conducted two numerical studies (Figure 4) using X-IGA in MATLAB [32] and the X-FEM in Ansys. Table 3 and Table 4 below outlines the preprocessing steps undertaken with the X-IGA method.

**Table 3:** X-IGA preprocessing data.

IGA Model with Enriched Control Points Near the Two Cracks	Boundary Conditions
Total number of control points = 1296 Uknot = [0 0 0 0.5 1 1 1]; Vknot = [0 0 0 1 1 1];	50 MPA stress applied in the upper side. Fixation in the lower side.

**Table 4:** Mesh refinement around the crack’s size (Figure 5, Figure 6, Figure 7 and Figure 8).

Coarse mesh size	3 mm
Mesh size of refinement 1	1 mm
Mesh size of refinement 2	0.33 mm
Mesh size of refinement 1	0.11 mm

### 4.3. Results and Discussion

This paragraph focuses on the post-processing stage of the numerical model simulated using the X-FEM and the X-IGA method, aimed at comparing T-stress, the SIF, and strain.

Recently, Ansys has emerged as a powerful software tool for crack propagation calculations, leveraging the new “SMART CRACK” functionality based on the X-FEM and the J-integral method. Its flexibility allows for the iterative refinement of the mesh around cracked zones without the need to repeat all of the preceding steps. The following table presents the T-stress results from the X-FEM simulation across varying mesh sizes (Table 5).

Graphically, the T-stress variation step decreases rapidly, which clearly shows the convergence of the T-stress value as a function of refining, and it is also an indicator of the reliability of the simulation, which can even replace experience to evaluate the X-IGA simulation.

**Figure 9:**
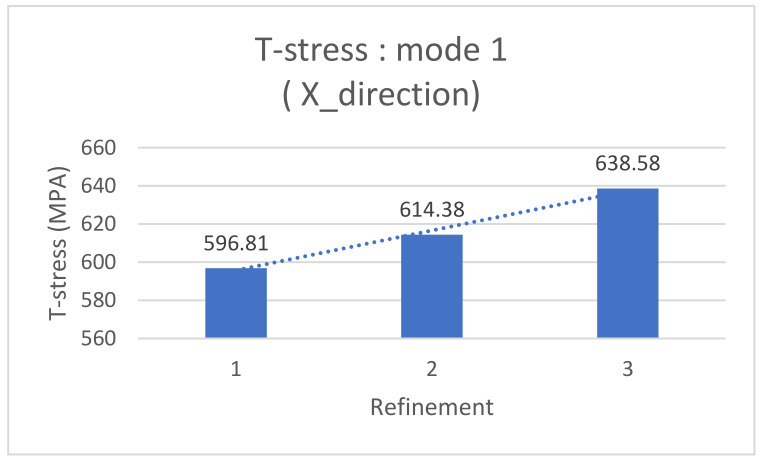
A graphical representation of T-stress in the first mode using X-FEM.

**Figure 10:**
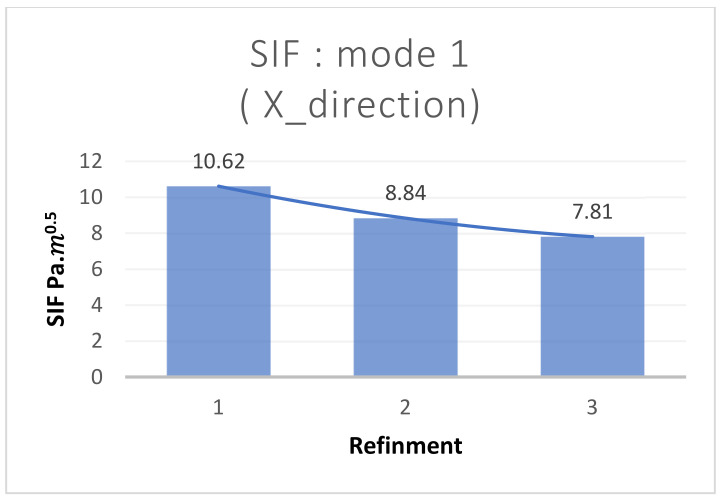
The graphical result for the SIF mode 1 using X-FEM in the function of refinement.

The Ansys 2021 software allows for the introduction of a damage study, enabling easy post-processing of stiffness intensity factors and other desired outputs. The authors have graphically plotted the curve showing the variation of the SIF with mesh refinement in Figure 9 and Figure 10, aiming to determine the optimal mesh size where convergence begins.

**Figure 11:**
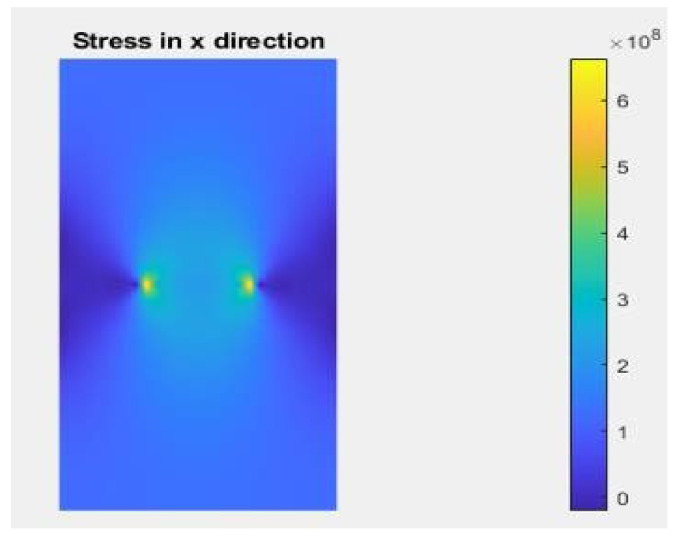
T-stress using X-IGA post-processing.

The novelty of this article lies in developing an IGA version through a MATLAB code with the same enrichment capability as the X-FEM, specifically for application with anisotropic materials. The post-processing of this simulation, along with IGA input data, is described in Table 6. It is noteworthy that the obtained values closely approximate those from the X-FEM, lending reliability to the results without the need for experimental validation (Figure 11). This hypothesis is further supported by the consistency observed between numerical and experimental results in articles [10,20].

The comparison of the maximum SIF and the tensile strain obtained by the two methods also confirms the approximate nature of the results (Table 7).

The maximum values of the SIF obtained from each simulation are described in the following Table 8. 

The results of tensile stress, deformation, and the maximum stress intensity factor (SIF) obtained from the two numerical simulations underscore the robustness of this study and bolster the hypotheses put forth in articles [16,33], which demonstrate the efficiency of X-IGA and the X-FEM by comparing results with experimental and analytical findings. With an extensive array of investigations at their disposal, the authors confidently assert the conformity and reliability of their findings. However, within this confirmation lies a crucial and pressing question: which methodology ultimately demonstrates superiority in predicting crack growth within composite anisotropic materials—X-IGA or the X-FEM?

Delving deeper into the realm of research, the studies outlined in papers [16,33,34] and [20] bring to light the effectiveness and versatility of non-uniform rational B-splines (NURBS) basis functions in accurately describing curved shapes. This stands in stark contrast to the traditional Lagrange polynomial interpolation method, which heavily relies on polygonal approximations and often necessitates a considerable number of elements to adequately capture the intricacies of curved geometry. One of the inherent strengths of NURBS functions lies in their adeptness at representing elliptical crack geometry with minimal control points, thus streamlining the computational process while maintaining precision and accuracy. In a seminal study conducted by S. Montassir and K. Yagoubi [8], it was demonstrated that X-IGA consistently yields SIF results with lower error rates compared to its X-FEM counterpart, all while employing fewer computational nodes and significantly reducing overall computational times. Moreover, the findings presented in the current article [35] further bolster this assertion, showcasing evidence within its tables that IGA not only decreases computation time but also reduces the number of Degrees of Freedom (DOFs) in finite element method simulations.

## 5. Conclusions

Basically, this paper’s main goal is to carefully investigate and compare the performance of X-IGA with the well-known X-FEM method in terms of determining the maximum stress intensity factor and tensile stress for the purpose of predicting crack growth within composite anisotropic materials. 

Moreover, it is critical to recognize the dominating trend in numerical simulations of fracture formation, which primarily concern orthotropic materials. Thus, starting with the creation of governing equations based on the elastic behaviour law for composite anisotropic materials and Stroh’s formula [11], the authors have attempted to expand the applicability and utility of such methodologies.

Through this initiative, the authors have been able to simplify the algorithmic framework that we used to update the X-IGA simulation’s code, using the workflow of Figure 3. The authors list some important findings and suggestions as follows:

- The numerical simulation process used in X-IGA is significantly faster than that of its FEM equivalent. This is mainly because control points are directly used, and the stiffness matrix is constructed using NURBS basis functions.
- The smooth transition from Computer-Aided Design (CAD) to the solution stage is made possible by this seamless integration, which eliminates the need for intermediate preprocessing tools and streamlines the computational workflow.
- The research suggests a new and straightforward design strategy to improve X-IGA’s usability and accessibility for composite anisotropic materials by using just the neutral to simplify the reduction of the three-dimensional (3D) and two-dimensional (2D) representations, which makes it easier to pre-process with X-IGA code and two-dimensional code.
- The convergence of the X-FEM results in the function of refining demonstrates the reliability of the results and can replace the experimental study to test the efficiency of the proposed X-IGA approach.

Expanding on the results from [35], it is clear that X-IGA is superior to the X-FEM, especially when it comes to smaller SIF mistakes and fewer control point needs. This benefit is mostly due to the following:

- NURBS functions are naturally precise and adaptable when it comes to approximating curved geometries; therefore, there is no need for significant modification.
- The Lagrange function’s approximation of curved geometry frequently requires significant refinement, which increases the need for elements and nodes in polygonal approximations and lengthens runtime, particularly when refinement is needed around a crack.
- This query highlights how dynamic and constantly changing numerical simulations are, and, as such, it is worth investigating further in the continuing effort to improve computational techniques in the fields of engineering and materials science.
- According to [36,37], X-IGA with few control points shows smaller SIF errors than the X-FEM, which can be attributed to NURBS functions’ accuracy in approximating curved geometries [38]. NURBS are able to accurately represent curved form behaviour in addition to approximating the true geometry. On the other hand, using Lagrange functions to approximate curved geometry necessitates a significant amount of refining [34], which raises the element and node requirements for polygonal approximations [39], which in turn lengthens runtime, particularly when refinement is required around a crack.

The main queries raised in our paper are: Why do we keep using the FEM rather than IGA? Could the FEM eventually be replaced by IGA?

As for the perspective of this work, we aim in the near future to explore the isogeometric analysis (IGA) method to conduct 3D calculations and demonstrate its capability to yield better results than the traditional finite element method (FEM). Additionally, we will investigate the optimization methods already employed within our teams [40,41,42,43,44,45] to mitigate crack propagation.

Even if IGA is better than the X-FEM in terms of precision and time optimization, it is not powerful and efficient for the contact modelling, so another investigation that we will work on with our team is how to combine the FEM and IGA to improve this field and introduce friction and contact interfaces.

## Figures and Tables

**Figure 5:**
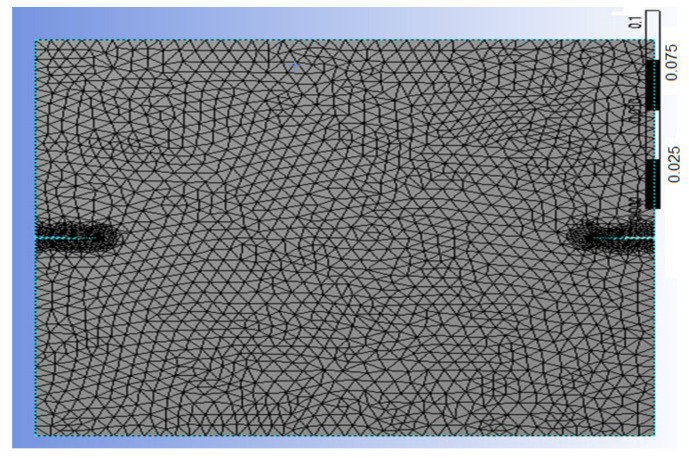
A meshed box (refinement 3).

**Figure 6:**
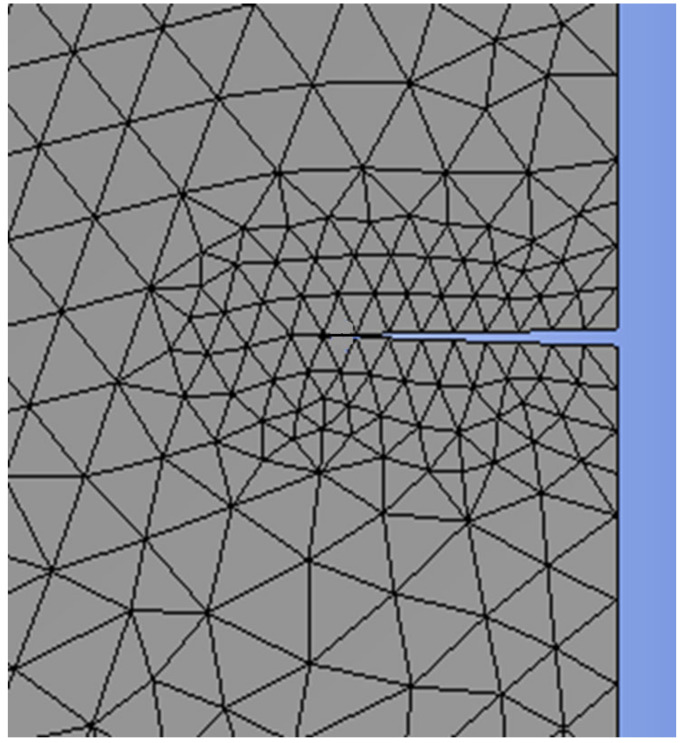
The X-FEM mesh refinement 1 around the right crack.

**Figure 7:**
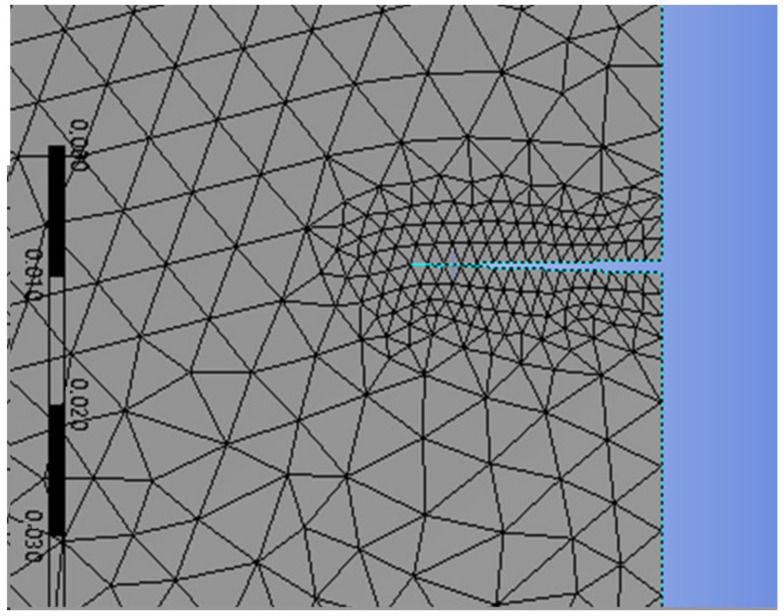
The X-FEM mesh refinement 2 around the right crack.

**Figure 8:**
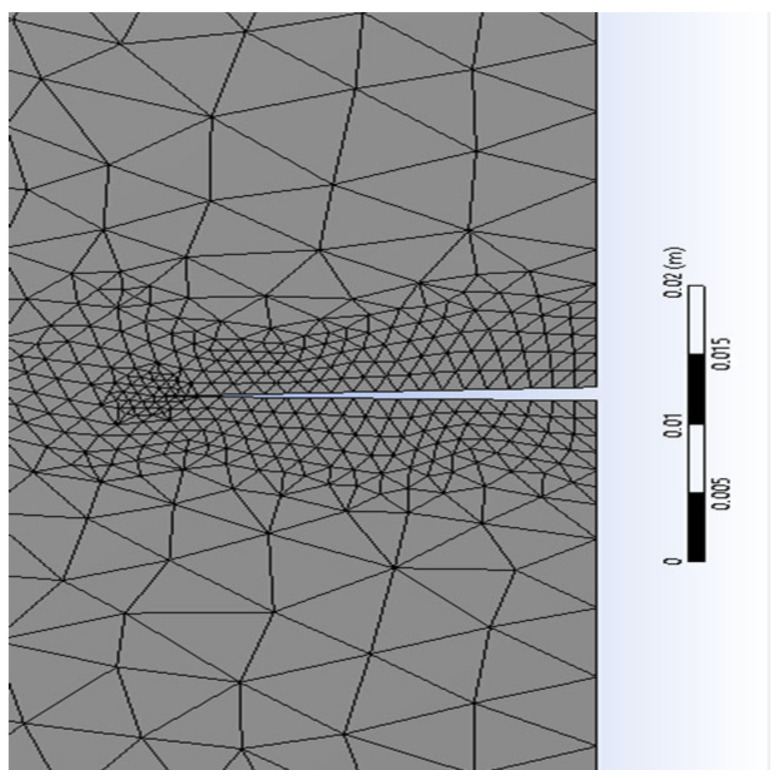
The X-FEM mesh refinement 3 around the right crack.

**Table 1:** The geometry inputs data.

Length	Thickness	Initial Crack Length: a
200 mm	2 mm	20 mm (two crack on the middle)

**Table 2:** The assigned material.

Material	Young Modulus in X Direction [GPa]	Young Modulus in Y Direction [GPa]	Poisson Coefficient XY	Coulomb Modulus XY [GPa]
Orthotropic composite material	121	8.6	0.27	4.9

**Table 5:** T-stress and the SIF post viewing, using the X-FEM in the function of refinement.

Number of Refinement around Cracks	SIF in Mode 1 $({\times 10}^{5}$$\mathrm{Pa}\cdot m^{0.5}$)	T-Stress (MPa)
1	10.62	596.81
2	8.84	614.38
3	7.814	638.58

**Table 6:** T-stress post viewing using X-IGA with h-refinement.

X-IGA Type of Refinement	h-Refinement
Maximum value of T-stress	638.58 MPa

**Table 7:** Strain in the x direction post viewing, using X-IGA and the X-FEM.

Numerical Approach	X-FEM	X-IGA
Maximum value	0.0206	0.0209

**Table 8:** The maximum stress intensity factor in mode 1 using the X-FEM and X-IGA.

Numerical Approach	X-FEM	X-IGA
Maximum stress intensity factor	7.814 × 10^5^ Pa·m^0.5^.	7.835 × 10^5^ Pa·m^0.5^.

## Data Availability

Data are contained within the article.
